# The Standard Scrapie Cell Assay: Development, Utility and Prospects

**DOI:** 10.3390/v7010180

**Published:** 2015-01-16

**Authors:** Jacques van der Merwe, Judd Aiken, David Westaway, Debbie McKenzie

**Affiliations:** Centre for Prions and Protein Folding Diseases, University of Alberta, Alberta, T6G2M8, Canada; E-Mails: jqvander@ualberta.ca (J.M.); jmaiken@ualberta.ca (J.A.); david.westaway@ualberta.ca (D.W.)

**Keywords:** standard scrapie cell assay, prion

## Abstract

Prion diseases are a family of fatal neurodegenerative diseases that involve the misfolding of a host protein, PrP^C^. Measuring prion infectivity is necessary for determining efficacy of a treatment or infectivity of a prion purification procedure; animal bioassays are, however, very expensive and time consuming. The Standard Scrapie Cell Assay (SSCA) provides an alternative approach. The SSCA facilitates quantitative *in vitro* analysis of prion strains, titres and biological properties. Given its robust nature and potential for high throughput, the SSCA has substantial utility for *in vitro* characterization of prions and can be deployed in a number of settings. Here we provide an overview on establishing the SSCA, its use in studies of disease dissemination and pathogenesis, potential pitfalls and a number of remaining challenges.

## 1. Introduction

Prion diseases, also known as transmissible spongiform encephalopathies (TSEs), are a closely related group of fatal neurodegenerative disorders affecting humans and other mammals [1,2]. They include Creutzfeldt-Jakob disease (CJD) in humans, bovine spongiform encephalopathy (BSE) in cattle, scrapie in sheep and chronic wasting disease (CWD) in deer, elk and moose. The etiological agent is PrP^Sc^, a misfolded form of the endogenous prion protein (PrP^C^) [2,3,4]. In prion disease, PrP^C^ is converted from its normal, predominantly alpha-helical structure to an aggregated, beta-sheet rich and largely protease-resistant form, PrP^Sc^ [5,6]. The newly formed PrP^Sc^ can further seed PrP^C^ to PrP^Sc^ conversion as the disease progresses.

The gold standard for establishing infectivity and titre of a prion infection is the animal bioassay, prototypically via intracerebral inoculation. Animal bioassays allow researchers to determine the incubation period (time to onset of clinical disease) and prion titre [7]. Prion titre is most accurately determined by end-point dilution in which a sample is sequentially diluted 10-fold and then a set of animals is inoculated with each of these serial dilutions. The end-point is when half the animals for a given dilution succumb to prion disease, expressed as the LD_50_ of a prion sample [7].

Western blotting is used in conjunction with animal bioassay to further characterize protease resistant PrP^Sc^. Routine Western blot analysis, however, is limited by its sensitivity (detection limit of 10^3^–10^4^ dilution of brain homogenate) [8,9], although higher-sensitivity versions have been developed [10]. Levels of protease-resistant PrP detected by Western blot analysis or any other assay do not necessarily correlate with infectivity levels [9].

Other alternatives to animal bioassay include both cell-based systems and cell-free methodologies. Although cell lines, in general, are not permissive to prion infection or replication. Several cell lines, most notably N2a cells [11,12,13], have been used to study prions *in vitro* (Table 1). Even infectible cell lines are not necessarily permissive to infection by all prion strains. For example, the N2a cell line is resistant to prion strains ME7, 22A and 301V [12,14] but susceptible to RML and 22L [12,14]. Several other *in vitro* assays have also been developed to bridge the gap between Western blot analysis of protease-resistant (*i.e.*, misfolded) PrP and *in vivo* animal bioassay; these include protein misfolding cyclic amplification (PMCA) [15], real time quaking-induced conversion (RT-QuIC) [16] and standard scrapie cell assay (SSCA).

### PrP^Res^ Amplification Methods

Saborio *et al.* developed a technique for amplifying PrP^Sc^ in a test tube, a process referred to as PMCA [15]. In PMCA, an infectious seed (PrP^Sc^) is incubated with template PrP^C^ [17,18,19]. Through a series of repeated incubations and sonications, the PrP^Sc^ signal is rapidly increased as PrP^C^ substrate is converted to PrP^Sc^ [15,20]. During the first phase (low PrP^Sc^ and excess PrP^C^), PrP^Sc^ is incubated with PrP^C^ to initiate PrP^Sc^ multimer formation [15,20]. During the second phase, the sample is sonicated breaking up the multimers, thereby revealing and increasing sites of PrP^Sc^ conversion [15,20]. The end result is a substantial increase in the number of seeds and a subsequent exponential increase in PrP^Sc^ formation [15,20]. PMCA results are obtained within days to weeks with PMCA product detection typically involving Western blot. PrP^Sc^ generated through PMCA shares similar structural and biochemical properties as PrP^Sc^ generated in an animal bioassay and is infectious in animal bioassays [20,21,22,23]. PMCA can be used to analyze low titre samples, examine species-barrier effects and evaluate the role of potential cofactors (polyanionic components, lipids and proteoglycans [1,24,25,26]) involved in PrP^Sc^ generation [21,27,28]. PMCA analysis of samples can, however, be confounded by the *de nova* generation of PrP^Res^ via off-target amplification of normal non-infectious material [29]. PMCA, therefore, requires a careful evaluation of controls to rule out possible false-positive results.

**Table 1:** Cell lines permissive to prion infection.

Cell Designation	Species	Tissue or Cell of Origin	Prion Strain	References
**Neuronal**				
N2a	Mouse	Neuroblastoma	Chandler, RML, 139A, 22L, C506, Fukuoka-1, FU CJD	[11,12,30,31]
GT1	Mouse	Hypothalamic	Chandler, RML, 139A, 22L, FU CJD, M1000	[30,32]
SMB	Mouse	Scrapie-infected mesodermal cells	Chandler, 139A, 22F, 79A	[30,33,34]
SN56	Mouse	Cholinergic septal cells	Chandler, ME7, 22L	[35]
CAD	Mouse	Catecholaminergic	RML, 22L, ME7, 301C	[14]
PC12	Rat	Pheochromocytoma	139A, ME7	[36,37]
**Non-Neuronal Cell Lines**				
C2C12	Mouse	Myotubes	RML, ME7, 22L	[38,39]
NIH/3T3	Mouse	Fibroblast	22L	[40]
moRK13	Rabbit	Epithelial cell line expressing mouse PrP^C^	22L, Chandler, M1000, mo sCJD, Fukuoka-1	[41,42]
voRK13	Rabbit	Epithelial cell line expressing vole PrP^C^	vole-adapted BSE	[41]
ovRK13/ RoV9	Rabbit	Epithelial cell line expressing ovine PrP^C^	PG127, LA404, SSBP/1, scrapie field isolates	[43,44]
**SSCA Cell Lines**				
PK1	Mouse	N2a	RML, 22L	[45,46,47]
R33	Mouse	N2a	RML, 22L	[14,46]
CAD5	Mouse	CAD	RML, 22L, ME7, 301C	[14,46]
LD9	Mouse	L929	RML, 22L, ME7	[14,46]
L929	Mouse	Fibroblast	RML, 22L, ME7	[40]
Elk21-	Rabbit	RK13: epithelial cell line expressing elk PrP^C^	CWD	[48]

Another sensitive PrP^Sc^ detection assay is quaking-induced conversion (QuIC) [16]. This cell-free conversion assay utilizes recombinant PrP^C^ (rPrP^C^) as a template and allows for the rapid conversion of rPrP^C^ to protease-resistant rPrP^Res^ [16]. This assay does not generate significant levels of infectivity and is, thus, suited to both the analysis and diagnosis of clinical samples as the reaction end-products are not an amplification of input titre [16,49,50]. In place of sonication used in PMCA, QuIC utilizes automated tube shaking [16,49,50]. QuIC allows detection of samples containing low levels (even subclinical) levels of prion infectivity. QuIC has been used to detect low levels of PrP^Sc^ present in cerebrospinal fluid (CSF) samples from both 263K-infected hamsters and scrapie-infected sheep [16,50]. Furthermore, QuIC requires a single day to perform, is more cost effective and easier to perform than PMCA [16,49,50]. Modifications of the QuIC assay facilitate the analysis of rPrP^Res^ formation by in-well monitoring levels of thioflavin T (ThT) fluorescence and allow for rPrP^Res^ detection within 24 h [49]. Referred to as real-time QuIC (RT-QuIC) [49], this modified method has been used to detect PrP^CJD^ in CSF samples from sCJD patients and has >80% sensitivity and 100% specificity when compared to control CSF samples [49]. RT-QuIC has also applied to the detection of scrapie in sheep, CWD in deer as well as rodent-adapted prions [51,52]. Although capable of detecting extremely low levels of PrP^Sc^, RT-QuIC is limited by its inability to study anti-prion compounds, decontamination methods and cellular process involved in PrP^C^ to PrP^Sc^ conversion since the final product appears to be non-infectious [53].

## 2. The Standard Scrapie Cell Assay

A crucial limitation of the cell-free conversion systems involves their inability to directly measure infectivity. An *in vitro* infectivity assay with the sensitivity of animal bioassay but at a fraction of the cost and time would, therefore, be advantageous. Such an assay would facilitate high throughput analysis of prion infectivity and allow the screening of libraries of potential anti-prion compounds. To this end, KlÖhn *et al.* established the Standard Scrapie Cell Assay (SSCA) in which cell cultures are infected with prions and then the PrP^Sc^-positive cells quantified [45,46]. PrP^Sc^-positive cells can be correlated with the starting material’s prion titre similar to an animal bioassay [45,46]. Over the past decade, the SSCA has been further modified providing a valuable assay for addressing a number of prion research questions; aspects of the assay are considered in the following sections.

The SSCA, as first developed by the Weissmann lab [45], was initially used to describe, discriminate and titre mouse prion strains using an N2a neuroblastoma cell subline, PK1 [45,46]. One common characteristic of cell line susceptibility is a clonal variability in ability to maintain prion replication [54]. The N2a neuroblastoma cell line requires substantial subcloning to obtain a highly susceptible line (N2a -> N2aPD88 -> N2aPK1) [45]. Although a number of cell lines have been used for the study of prions *in vitro* (Table 1), not all of these are viable options for the SSCA (Table 1) and typically require significant subcloning to obtain a highly susceptible (to prion infection) subline. While the majority of cell lines (Table 1) used in the SSCA are susceptible only to murine prion agents [45,46]. RK13 cell lines (derived from rabbit kidney epithelial cells) [41] transfected to express either cervid or ovine PrP^C^ has been used to study CWD [48] and sheep scrapie isolates [55], respectively. Cell lines susceptible to infection by BSE or CJD are not currently available.

### 2.1. Establishing the Standard Scrapie Cell Assay

**Figure 1:**
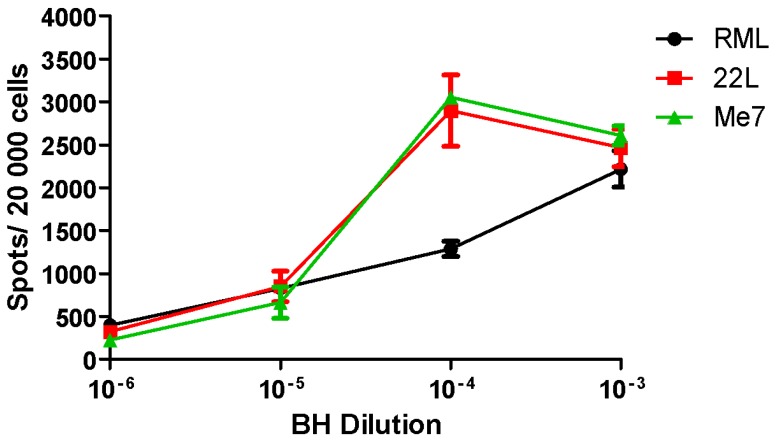
SSCA of RML, 22L and ME7 in L929 Cells. L929 cells were exposed to serial dilutions of RML, 22L and ME7. The generation of PrP^Sc^ was determined by ELISPOT analysis following three passages of the cells. L929 cells demonstrated a dose-dependent PrP^Sc^ spot response to all three prion strains with 22L and ME7 generating the most signal. *N* = 6, mean ± SEM.

Four cell lines have predominantly been used in the SSCA: PK1, CAD5, R33 and LD9 (Table 1). PK1 and R33 cells are derived from the N2a neuroblastoma cell line [14,45], CAD5 cells are a subclone of the murine Cath.a differentiated cell line [14], while LD9 cells are a subline of the mouse fibroblast L929 cell line [14]. L929 cells have been demonstrated to be susceptible to prions [40] and thus posed as a good candidate for use in the SSCA. Guided by reasons discussed below we have thus far adapted the SSCA for use with both the L929 mouse fibroblast cell line (Figure 1) and RK13 cells (rabbit kidney epithelial cells) stably transfected with mouse PrP^C^ (RK13moPrP).

**Figure 2:**
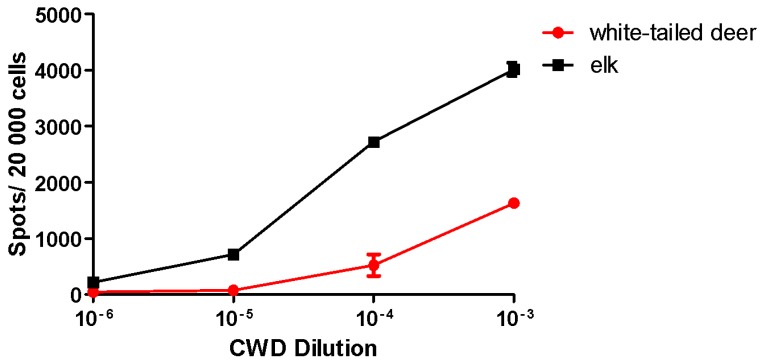
CWD Isolate Titration in Elk21- Cells. Elk21- cells were exposed to serial dilutions of two different CWD isolates (elk, 132M, and white-tailed deer, 95Q/96G) and the generation of PrP^Sc^ determined by ELISPOT analysis following three passages of the cells. Elk21- cells demonstrated a dose-dependent PrP^Sc^ spot response to elk and white-tailed deer CWD. *N* = 6, mean ± SEM.

Cell lines used in the SSCA exhibit distinct susceptibility to prion strains (Table 1). Similar to LD9 cells [14], L929 cells generate PrP^Sc^ in response to 22L, ME7 and RML prions (Figure 1). Importantly, these results demonstrate comparable responses to the already published LD9 subline [14,45]. In contrast, PK1 [14] and RK13moPrP cell lines generate PrP^Sc^ in response to RML and 22L prions but not ME7. While the SSCA was initially limited to murine cell lines infected with murine prions, a non-murine cell line compatible with the SSCA is the recently developed elk PrP^C^-expressing RK13 cell line (Elk21-) [48]. Based on a report demonstrating retroviral Gag-enhanced release of PrP^Sc^ from scrapie-infected cell lines [56], the elk PrP^C^ RK13 line was further transfected with pcDNA3-gag, expressing the HIV-1 GAG precursor protein. This resulted in a cell line that, upon infection with elk CWD, exhibited an ~2-fold increase in PrP^Sc^ generation [48,56]. The Elk21- cell line generates PrP^Sc^ in response to both elk and white-tailed deer CWD [48], the latter generating significantly fewer PrP^Sc^-positive cells compared to elk CWD [48] (Figure 2). The development of additional cervid cell lines will, therefore, help to further evaluate various CWD isolates from both deer and elk. To the best of our knowledge, there are no cell lines currently available that can be infected with primary isolates of BSE or CJD prions.

### 2.2. Utility and Sensitivity

#### Prion Strain Titration, Discrimination and Analysis

Distinct strains of prions—“true-breeding” isolates—can exist in animals of the same host genotype [57,58,59,60,61]. Prion strains can be distinguished by clinical, pathological and biochemical characteristics including incubation time before disease onset, the severity of spongiform change in different neuroanatomical regions of grey and white matter (“lesion profiles”) and the areas of deposition of aggregated PrP^Sc^ detected by immunohistochemistry [7,62,63]. Furthermore, prion strains can exhibit distinct biochemical and biophysical properties, including PrP glycoform profiles, protease resistance, protease cleavage sites as well as denaturation profiles [28,64,65,66].

The SSCA can be used to distinguish prion strains based upon the strain response in a given cell line. For examples, RML, 22L, ME7 and 301C prion agents can be differentiated on their ability to infect a panel of cell lines: PK1, R33, CAD5 and LD9 [14]. Similarly, we found that L929 cells (mouse fibroblast cells) also distinguish between the different murine prion strains analogous to LD9 cells [14,45,46]. In both L929 cells and the LD9 subline, 22L and ME7 generate the most PrP^Sc^-positive cells with RML generating the fewest (Figure 1). Increasing the amount of input PrP^Sc^ BH (22L and ME7) further enhances the PrP^Sc^ generated by L929 cells in the SSCA until it plateaus, *i.e.*, the 10^−4^ and 10^−3^ dilution of input BH result in the largest number of PrP^Sc^-positive cells and a plateau. RML, however, does not appear to reach a plateau in L929 cells. The different responses observed in RML, 22L and ME7 treatment illustrate an important aspect of the SSCA. Comparing different strains or isolates requires a standardized titred control given that different strains or isolates may generate varied PrP^Sc^ levels within a given cell line, *i.e.*, ME7 in LD9, L929 compared to no response in PK1 cells [14,45]. In the presence of N-glycosylation inhibitors (swainsonine (inhibits Golgi-mannosidase II), kifunensine (inhibits α-mannosidase I) and castanospermine (inhibits α-glucosidase), the SSCA can be used to assess strain differences. For example, swainsonine inhibits the infection of PK1 cells by RML, 79A and 22F [64] while exhibiting a smaller effect on 139A and having no effect on 22L in PK1 cells. Interestingly, swainsonine has no effect on any of these four strains in either CAD5 or LD9 cells [64].

Transfected RK13 cells are, as noted above, permissive to infection by prions from several murine strains (RML, 22L), from bank voles, natural isolates of scrapie and from elk CWD (Table 1) [41,48,55]. A cervid prion cell assay (CPCA) was recently developed using Elk21- cells [48]. We have used the Elk21- cells to assay elk and white-tailed deer CWD isolates (Figure 2). Elk CWD generates a significantly more robust response compared to white-tailed deer CWD (Figure 2). Interestingly, compared to the murine strains, the elk and white-tailed deer CWD isolates do not appear to plateau. With both the elk and white-tailed deer CWD, the 10^−3^ dilution generates with most PrP^Sc^, with the 10^−4^ dilution generating a smaller amount. Significant differences between PrP^Sc^ generation at various BH dilutions help improve the dynamic range of the SSCA where one is able to distinguish and characterise a wider range, *i.e.* 10^−6^ to 10^−3^ for elk CWD and 10^−5^ to 10^−3^ for white-tailed deer CWD. Given the substantial *Prnp* sequence homology between elk and white-tailed deer (a single difference at position 226), these data confirm that small changes in the *Prnp* sequence correlate with different potential infectivity outcomes (e.g., lower CPCA response) based upon the host *Prnp* sequence [67,68,69].

Routine analysis of samples using the SSCA can approximate changes in titre of the inocula (*i.e.*, by comparison of the spot count of a “test sample” compared to the standard curve generated by titred agent). Although endpoint titrations are normally determined by animal bioassay, a modification of the SSCA allows endpoint titration, generating titres that correlate with animal bioassay data but at a fraction of the cost and time [45,46].

## 3. Anti-Prion Compounds and Drugs

Currently, there is no cure and no effective treatment for prion disease. As such, the development of anti-prion compounds will be critical in battling this fatal neurodegenerative disease. A number of anti-prion compounds have previously been described, however, while these compounds can be highly effective *in vitro*, the majority of them have little to no effect *in vivo* [70]. Identification of potential *in vivo* candidates involves screening of thousands of compounds, thus, a high throughput *in vitro* platform is required. Using known anti-prion agents, we have demonstrated that SSCA has the potential to become a high throughput screening platform.

**Figure 3:**
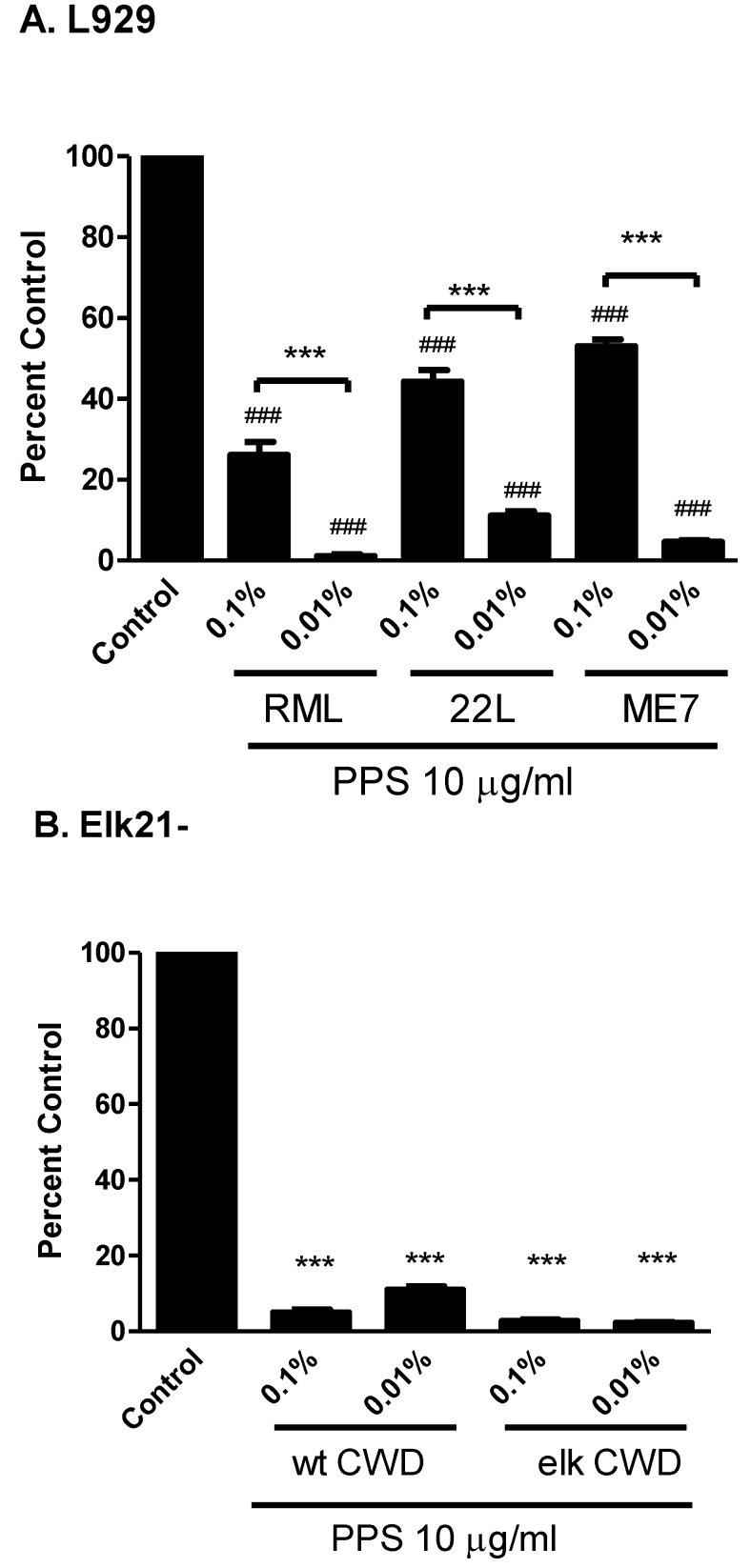
Pentosan Polysulphate Inhibition of PrP^Sc^ in L929 and Elk21- Cells. (**A**) L929 cells were exposed to serial dilutions of RML, 22 L and ME7 in the presence of 10 μg/mL pentosan polysulphate (PPS). PPS significantly attenuated PrP^Sc^ generation in both 0.1% and 0.01% RML, 22L and ME7 brain homogenate (BH) treatments compared to control (### *p* < 0.001). PPS displayed a significantly greater inhibitory effect in the 0.01% BH treatments compared to the 0.1% BH treatments (*** *p* < 0.001). Control: RML 0.1% BH; (**B**) Elk21- cells were exposed to serial dilutions of 2 different CWD isolates (elk; 132 M, and white-tailed deer; 95Q/96G) in the presence of 10 μg/mL PPS. Control: Elk or white-tailed deer CWD 0.1% BH. The generation of PrP^Sc^ was determined by ELISPOT analysis following three passages of the cells. PPS significantly attenuated PrP^Sc^ spot generation in Elk21- cells when compared to control (*** *p* < 0.001 *vs.* control). There was no significant difference between 0.1% and 0.01% CWD BH in the presence of PPS. *N* = 6, mean ± SEM.

A standard assessment of potential anti-prion compounds assayed in chronically infected cells involves PK digestion and PrP^Sc^ visualization by Western blot. This approach significantly limits the number of compounds that can be screened. SSCA, on the other hand, can be used to screen hundreds of compounds with relatively high-throughput [14,45,46]. Pentosan polysulphate (PPS), a known anti-scrapie drug, can with continual treatment extend incubation time in rodent models of prion infection. We examined the effectiveness of PPS (10 µg/mL) in the prevention of PrP^Sc^ generation in both L929 (RML, 22L, ME7) and Elk21- (white tailed deer and elk CWD) cells (Figure 3A and B, respectively). PPS significantly reduced the PrP^Sc^ generation in L929 at both 0.1% weight/volume (w/v) and 0.01% w/v prion agent dilutions, although the observed inhibition was significantly lower at 0.01% w/v (Figure 3A). In Elk21- cells, the PPS was equally effective at reducing PrP^Sc^ generation in response to both white-tailed deer and elk CWD when using either 0.1% w/v or 0.01% w/v prion agent.

The SSCA has also been used to determine the ability of synthetic polymers (dendrimers) to reduce prion infectivity *in vitro* [71]. The anti-prion effectiveness of mPPIg5 (dendrimer) and two other known anti-scrapie compounds, suramin and STI571, were demonstrated in PK1 cells [71]. The usefulness of the SSCA to identify potential anti-prion agents has also been demonstrated in a number of other studies; including the curing of chronically infected Elk21- cells by dextran sulfate [48] and chronically infected mouse cells by PPS [45,46]. Elk21+ and RKD+ cells were also utilized to study the effects of quinacrine using the SSCA [72]. Interestingly, quinacrine increased the number of PrP^Sc^-positive cells following their treatment for six days but prolonged incubation times in animal bioassay [72]. SSCA could provide a means for a relatively high throughput primary screen of a compound library with lead compounds targeted for further evaluation in other *in vitro* and, ultimately, *in vivo* assays.

## 4. Prion Infectivity and Decontamination Studies

The SSCA can also be used to quantify and measure the effectiveness of prion decontamination methods. Edgeworth [73] demonstrated drastic variations in the ability of commercially available decontamination methods to inactivate RML prions using the SSCA in the steel-binding format [73]. These studies examined the anti-prion effectiveness of chemical treatment on prions bound to steel wires. Prions avidly bind to metal surfaces, including steel [74]. For example, it is currently recommended that surgical instruments be autoclaved at 134 °C for 18 min, however, even when autoclaving at 134 °C for 15 min, steel wires exposed to high titres of prion infectivity (10^5.3^ LD50 units/mL, ~10^−4^ dilution of brain homogenate) continue to display significant infectivity [73]. Prion-coated steel wires can efficiently transmit prion disease to mice [75] and are also capable of infecting cell cultures [76]. This method of delivery has been adapted for SSCA [73]. This detection of extremely low titres of steel wire-bound prion samples has improved the dynamic range of the SSCA (detection limit of 10^−8^ compared to 10^−10^ in the steel binding assay) and allowed the assessment of vCJD blood samples [77].

Furthermore, combining the use of prion-coated steel wires with the SSCA facilitates the quantitative assessment of the effectiveness of prion decontamination procedures [73]. Of the reagents tested by Edgeworth *et al.*, Rely + On PI (DuPont Corporation), Prionzyme (Genencor) and 2 M NaOH appeared to be the most effective. Of note, Prionzyme is prepared in 2 M NaOH and gives indistinguishable results from 2 M NaOH alone [73]. The study found the HAMO 100 PID reagent (0.8% and 1.6%) to be ineffective at decontaminating RML-coated steel wires [73]. The SSCA, when used in the steel binding assay format, could play a critical role in developing new strategies for decontaminating steel surfaces and surgical instruments.

## 5. Detection of Infectivity from Environmental Samples

Environmental CWD contamination has the potential to present a significant reservoir for the oral transmission of CWD prions. CWD remains infectious in the environment for substantial periods of time and may potentially do so by binding to soil particles [78,79,80]. CWD prions are present in the blood, placenta, urine, saliva and feces of infected deer [78,81,82,83] thereby adding to the potential for environmental contamination. A study by Genovesi *et al.* examined prion-exposed soil samples (arable sandy-loam and quartz sand) in the SSCA using PK1 cells [84]. Prions adsorbed onto arable sandy-loam and quartz sand remained infectious [84]. These results are similar to those obtained by animal bioassay where Syrian hamsters were inoculated with PrP^Sc^ bound to montmorillonite and it remained infectious [85]. RML-bound quartz sand particles generated a greater PrP^Sc^ signal when compared to arable sandy loam [84]. Although the SSCA has been applied to the study of prions in environmental samples, these studies pose significant remaining challenges. Exposing the cells to environmental samples, e.g., soils or soil components, may have adverse toxic effects on the cells affecting the SSCA readout.

## 6. SSCA Materials and Methods

**Figure 4:**
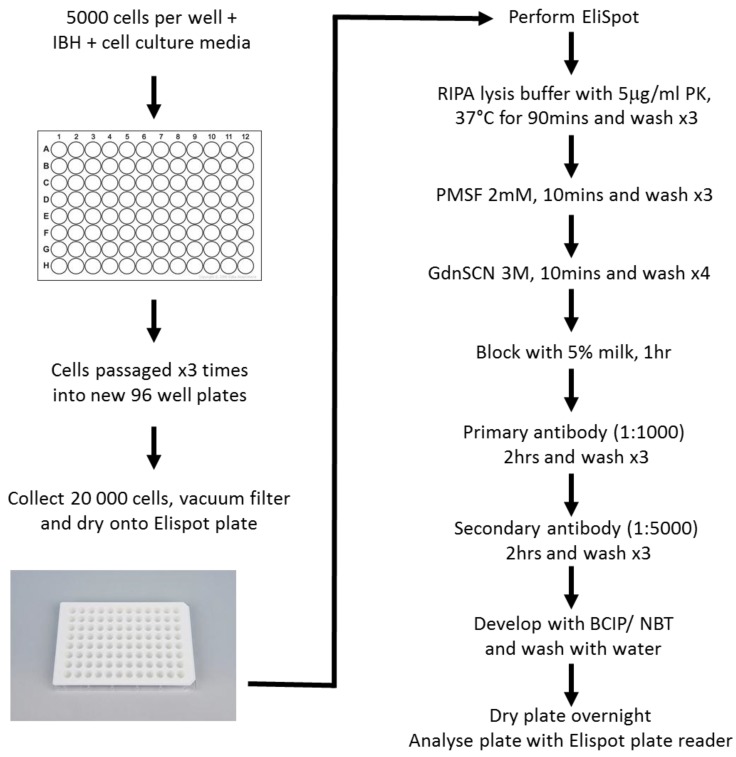
Flow Chart of the Standard Scrapie Cell Assay.

The SSCA employs a prion-infectible cell line, which is exposed to serial dilutions of infectious brain homogenate (IBH) in a 96-well plate (Figure 4). The cells are grown for 4–5 days, passaged 3 times and then 20,000 cells are filtered onto an Elispot plate for analysis. The SSCA response is a measure of the number of PrP^Sc^ positive cells and is indicative of the original starting infectious titre [45,46]. The SSCA has a standard error of 15%–25%, can be as sensitive as a mouse bioassay (depending on the cell line used), is substantially more rapid than bioassay (weeks *vs*. months or years), cheaper, and capable of high throughput analysis with automated liquid handling steps [45,46].

Our SSCA protocol is based on a modification of a procedure initially described by Klöhn *et al.* [45,46]. Serial dilutions of infectious brain homogenate are pipetted into a 96-well cell culture plate. L929 cells (5000 cells) are added to the plate and allowed to incubate 3–5 min with the infectious material prior to the addition of cell culture media (DMEM containing 10% horse serum). The ratio of prion infectivity to the total number of cells is a critical factor (analogous to a multiplicity of infection in a viral plaque assay) where an optimal PrP^Sc^ infectivity unit to cell number ratio is required. If this ratio is too low, the infection will likely not be established or the cells may outgrow the PrP^Sc^ conversion. If the ratio is too high, the brain homogenate may exert toxic effects on the cells, residual inoculum may persist at the end of the assay and/or the PrP^Sc^ signal may be too strong to accurately evaluate by Elispot. The cells are cultured for 5 days and passaged 1:4 following trypsinization, cultured an additional 5 days and passaged at 1:7. The cells are grown for another 5 days, harvested and 20,000 cells vacuum-filtered onto activated Elispot plates.

The SSCA can be modified and performed in an end point titration format (detection titres comparable to those in animal bioassay). In this modification, cells are passaged at least 12 times and the number of positive wells scored (mean value of spot count exceeding background by 5 SDs) [45,46]. The titre is then calculated based on the dilution at which 50% of the wells score positive. Endpoint titration of prion samples in the SSCA is currently limited to PK1 cells [46].

Another modification of SSCA includes the steel binding assay. For these modified SSCA experiments, PK1 cells are used to assay prion-coated steel wires (Steelex monofilament wires, USP 4/0, cut into 2.5 cm pieces and exposed to various infectious brain homogenate dilutions) at dilutions as low as 10^−10^ (equivalent to ~100 pg of RML prion-infected brain tissue per milliliter) [73]. This is approximately 100-fold more dilute than can be analyzed by animal bioassay [73]. Once prion-coated, the steel wires are washed and placed in a 6 well culture dish (20 wires per well) containing 300,000 PK1 cells for 3 days. Following the 3-day exposure, cells adhering to the wires are collected and assayed by SSCA [73].

Once the cells are vacuum-filtered onto an Elispot plate, the plate is dried at 50 °C for 1 h and stored at 4 °C. To continue processing, the plates are incubated with proteinase K (5 μg/mL; Roche Diagnostics) in RIPA lysis buffer (0.05 M Tris, pH 7.5, 0.15 M NaCl, 0.1% SDS, 1% NP-40, 1% deoxycholic acid) for 1.5 h. The plates are then washed, treated with 2 mM phenylmethanesulfonylfluoride, the PrP^Sc^ denatured by addition of 3 M guanidinium thiocyanate for 10 min and the membranes blocked for 1 h using 5% milk in Tris-buffered saline (TBS). Finally, the plates are incubated with an anti-mouse PrP^C^ antibody (SAF83, 1:1000 in TBS; Cayman Chemical) for 2 h, washed 3 times, incubated with goat anti-mouse alkaline phosphatase-conjugated secondary antibody (1:5000 in 1% milk TBS) for 2 h and extensively washed. The Elispot plate under-drain is then removed, the membranes blotted and alkaline phosphatase (AP) buffer added (10 min). The AP buffer is removed and the plates developed using 5-bromo-4-chloro-3-indolyl-phosphate and nitro blue tetrazolium for 20 min. Once developed, the plates are washed with distilled water, dried overnight in the dark and then analyzed using an AID Elispot plate reader (Autoimmun Diagnostika GmbH, Straßberg, Germany). Thresholds for the reader are: Intensity 4, Size 1 and Gradient 0 using algorithm B. Using the AID Elispot plate reader at these settings allows for an optimal background noise to signal ratio (mock-treated and control wells have minimal spot counts). Typically background noise is less than 30 spots. The same count and camera settings are used for all plates.

Analysis of the SSCA readout requires a number of controls. To readily determine titre of a sample, a stock of infectious agent that has been titred (initially by bioassay) is required for each prion agent and cell line combination. Mock-infected cells provide the background number of spots and allow the determination of the practical “cut-off” value for the assay [45,46]. Other important SSCA controls include an “inhibited control” (anti-PrP^C^ antibody is co-incubated with the sample to sequester substrate and, thus, prevent conversion of PrP^C^ to PrP^Sc^ (we have used the SAF83 antibody for this). The application of an authenticated, known prion replication inhibitor such as pentosan polysulfate at 10 μg/mL to prevent PrP^Sc^ generation is also an important control.

## 7. Limitations of the SSCA

There remain a number of caveats and ongoing challenges with the SSCA. Although a number of murine cell lines (PK1, R33, CAD5 and LD9) can be used in the SSCA, not all of these are widely available and, thus, the assay can be difficult to establish in cell panel format. Furthermore, as PK1 and R33 cells are N2a-derived, they are polyploid (containing roughly six copies of the PrP gene, for example) [86] and the expression of PrP^C^ is significantly variable between individual sublines, resulting in heterogeneous cell populations [14]. Due to this inherent variability within the N2a cell line and its propensity to lose susceptibility to prion infection with successive passaging, N2a-derived sublines are routinely maintained for only 8–10 passages [46]. In a typical SSCA, the maintenance stock is passaged a number of times and then the cells are passaged at least three more times during the assay, requiring that reference cell stocks continually be re-established [46]. The L929 cell line appears more stable in our studies (ability to be infected with prions is not lost) and capable of 20–30 passages prior to the requirement of new stock. L929 cells are, however, less sensitive to some murine prion strains, especially RML, than CAD5 or PK1 cells precluding endpoint titrations.

SSCA performed in an endpoint titration format is capable of assessing prion titres comparable to those assayed in animals. This format requires a cell line to be infected with prions and then continually be passaged (>10 passages) over a period of weeks. Therefore, a delicate balance between cell growth and prion replication is required. In the endpoint titration format, cells are exposed to an extremely dilute homogenate (~5–20 infected cells per 20,000) and passaged 12 times [46]. Due to specific cell line growth characteristics, the majority of cell lines will clear the infection as they continue to grow and divide, limiting their ability to establish chronic infections. Currently, endpoint titrations in the SSCA are limited to PK1 cells [46].

These cell sensitivity issues further illustrate the strain specificity of the currently available SSCA cell lines. Although a prion strain may be capable of infecting and replicating in a given cell line, the cells will likely require substantial subcloning to obtain a line that is highly susceptible to prion infection, e.g., PK1 from N2a cells, CAD5 from cath.a-differentiated cells and LD9 from L929 cells [14]. Subcloning of these cell lines led to the development of a panel of cell lines (PK1, R33, CAD5 and LD9) capable of assaying multiple murine strains (RML, 22L, ME7 and 301C) [14]. Similarly, L929 and RK13-mouse PrP^C^ cell lines exhibit distinct prion strain preferences. L929 cells generate a robust response to ME7, 22L and RML, while the RK13-mouse PrP^C^ line does not respond to ME7. These two lines could, therefore, be used to further characterize and distinguish different murine strains in the SSCA. Our findings and those presented by cell panel SSCA experiments [46] suggest that, beyond PrP^C^ expression, other factors contribute to the replication of prion strains *in vitro*. These may involve differential uptake or processing of prions and the expression of other host proteins or co-factors [30,87]. The study of prion strains and isolates that are not mouse-adapted, however, still poses a significant challenge in the SSCA.

The SSCA is exceedingly useful for analysis of murine-adapted strains. The utility for other prion strains is still somewhat limited due to the paucity of cell lines infectible with prion agents that are not mouse adapted. Currently two cell lines are available for the study of non-murine strains and isolates. These include RK-13 cells expressing ovine PrP^C^ (ovine scrapie) [41,88] and Elk21- cells (CWD) [48]. We have used the Elk21- cells to examine different CWD isolates (elk and white-tailed CWD). The Elk21- cells display distinct isolate preferences, with elk CWD generating a significantly larger SSCA response. These findings suggest that the Elk21- cells could be further utilized to study different CWD isolate *Prnp* polymorphisms. Unfortunately, no cell lines exist for the study of BSE or CJD.

The use of prion-coated steel wires has further expanded the utility of the SSCA. However, one unexpected consequence of the use of steel wires described earlier is the observation of spontaneous formation of prion infectivity (10^4.5^ LD_50_/mL of 100× conditioned media) from steel wires dipped into normal brain homogenates [89]. While this spontaneous conversion may ultimately shed light on a process that gives rise to sporadic CJD, it underscores a practical noise-floor issue and results should be carefully controlled as to avoid potential false positives. It should be noted that spontaneous formation occurs less frequently in SSCA compared to PMCA.

## 8. Summary and Conclusions

The SSCA, when implemented through the use of multiple cell lines such as PK1, L929, LD9, R33 and CAD5, can distinguish different strains of mouse-adapted prion isolates. Furthermore, quantitation of prion infectivity titres similar to those observed in bioassay in mice was obtained using the PK1 cell line. Thus the SSCA can be appreciated as a valuable research tool capable of addressing questions about prion strains and strain evolution, compound inhibitors and decontamination methods, while simultaneously reducing the need for animal bioassays. With the development of other infectible cell lines (ovine and cervid), the SSCA capabilities have been expanded to the study of other species; however, the development of cell lines susceptible to BSE and CJD remains a priority.
